# Comparison between the Efficacy of *Nigella sativa*-Honey and Clotrimazole on Vulvovaginal Candidiasis: A Randomized Clinical Trial

**DOI:** 10.1155/2022/1739729

**Published:** 2022-10-14

**Authors:** Masoumeh Norouzi Allahleh Korabi, Seyedeh Tahereh Mirmolaei, Malihe Tabarrai, Seyedeh Mojgan Ghalandarpoor-Attar, Seyede Nargess Sadati lamardi, Shima Haghani

**Affiliations:** ^1^School of Nursing and Midwifery, Tehran University of Medical Sciences, Tehran, Iran; ^2^Department of Midwifery and Reproductive Health, School of Nursing and Midwifery, Tehran University of Medical Sciences, Tehran, Iran; ^3^Department of Persian Medicine, School of Persian Medicine, Tehran University of Medical Sciences, Tehran, Iran; ^4^Department of Obstetrics and Gynecology, School of Medicine Baharloo Hospital, Tehran University of Medical Sciences, Tehran, Iran; ^5^Department of Traditional Pharmacy, School of Persian Medicine, Tehran University of Medical Sciences, Tehran, Iran; ^6^Nursing Care Research Center, School of Nursing and Midwifery, Iran University of Medical Sciences, Tehran, Iran

## Abstract

**Materials and Methods:**

This triple-blind, randomized controlled trial was conducted on eighty-four 18 to 49-year-old nonpregnant women from August 2019 to February 2020. The subjects were randomly divided into two groups after confirming the diagnosis of VVC infection through fungal culture. Clinical signs and symptoms and lab tests were recorded at baseline and 6–10 days after treatment. The treatment time for each group was seven nights.

**Results:**

There were no significant differences in clinical and laboratory evaluations between the two groups at the beginning of the study (*P* > 0.05). After treatment, secretion, redness, itching, and fungal culture improved in the two groups (*P* < 0.001), while pruritus (*p* = 0.013) and secretion (*p* = 0.025) in the control group significantly improved. In this trial, no patients showed drug-specific side effects.

**Conclusion:**

The results of this study show that the *N. sativa*-honey significantly improves the symptoms of VVC; thus, the application of *N. sativa*-honey can be considered as a complementary therapy in the treatment of VVC. This trial is registered with IRCT20190711044176N1.

## 1. Introduction

Vulvovaginal candidiasis (VVC) is one of the most common lower genital tract infections in women of childbearing age [1], affecting at least about three-quarters (75%) of women throughout their life [2]. Approximately 5–8% of women may develop recurrent vulvovaginal candidiasis (RVVC) [3]. RVVC is defined as 4 or more VVC episodes per year [3].

The incidence of VVC among symptomatic women was 12.1% to 57.3% [4]. This infection is caused by the abnormal growth of *Candida* yeast, which is a part of the normal vaginal flora [5] (changing from a typical round-ovid yeast cell (Y) to a hyphae mycelial growing organism (H)). Lactobacillus produces lactic acid, H_2_O_2_, and antimicrobial compounds to inhibit the transformation of *Candida* albicans Y to H type [2, 6].

Cottage cheese like secretions and vulvovaginal itching are common features of VVC infection. The vulvovaginal area may also experience burning sensations, erythema and edema, odorless discharge, and watery to thick vaginal discharge [7]. Although, these symptoms may also appear in many other vaginal infections [8].

VVC is usually diagnosed by common patient complaints and clinical examination, but microscopic examination of wet smears of patients (with normal saline and 10% potassium hydroxide) can also be diagnosed. Vaginal secretion culture is the standard method for confirming VVC and is used in cases with negative wet smears [9]. Women with VVC have a normal vaginal pH (4 to 4.5) [10].

The most common predisposing factors for VVC are recent antibiotic use, increased estrogen status (for example, high estrogen contraceptives, and pregnancy), hormone replacement therapy, poor diabetes control, wearing tight clothing, and sexual activity [11, 12].

The treatment of choice is an azole antifungal drug, such as clotrimazole. However, inappropriate use of these over-the-counter drugs has led to drug resistance, and subsequently greater prevalence of this infection [13, 14].

*Candida* albicans is responsible for 85% to 90% of VVC cases. *Candida* glabrata is accounted for almost all of the remaining cases. It is worth mentioning that in patients with *Candida* glabrata vaginitis, azole treatment failure is common (approximately 50%) [7, 9]. Therefore, given the resistance to azoles, it seems necessary to find natural and adequate alternatives to prevent or treat VVC. One herb used in VVC is *Nigella sativa*.

*N. sativa* L. (black seed and black cumin) from the Ranunculaceae family is a plant, which is widely used as a medicinal plant with antifungal properties and has been used around the world, especially in the Middle East to treat the symptoms of VVC [15, 16]. Most of the properties of this plant are related to thymoquinone [17, 18].

Another complementary treatment for VVC is honey. For many years, honey has played a special role in traditional medicine [19].

The combination of *N. sativa* and honey has long been used to treat various diseases [20]. Therefore, the combination of *N. sativa*-honey can be one of these complementary methods for the prevention or treatment of VVC. Due to N. sativa and honey have antifungal and antibacterial properties. In previous studies, the combination of *N. sativa*-honey has been successfully used to heal wounds [21].

Although some studies have evaluated the antifungal effects of *N. sativa* and honey alone, and the combined use in vitro and in vivo studies [17, 21, 22], no study has identified the effect of *N. sativa* and honey together through vaginal products at VVC. The therapeutic effect of each component alone has been proven in previous studies and the purpose of this study was to investigate the formulation mentioned in traditional medicine sources that *Nigella sativa* product is used with honey. Therefore, this randomized, triple-blind clinical study aimed to evaluate the effect of *N. sativa*-honey vaginal cream compared with clotrimazole vaginal cream on VVC in women of childbearing age.

## 2. Materials and Methods

### 2.1. Study Design and Setting

This study was a randomized controlled trial, triple-blind, two parallel groups, one intervention group (*N. sativa*-honey vaginal cream), and one control group (1% clotrimazole vaginal cream). The study aimed to compare the effects of the *N. sativa*-honey and clotrimazole on VVC among women referred to the Gynecology Clinic of the Baharloo Hospital in Tehran, Iran, from August 2019 to February 2020.

All eligible participants were thoroughly informed of the research and procedures, a consent form was taken before beginning of the trial from them. The study participants were confident that they can withdraw from the study at any stage of the trial. The research protocol was approved by the Ethics Committee of the School of Nursing, Midwifery, and Rehabilitation of Tehran University of Medical Sciences (no. IR.TUMS.FNM.REC.1398.074) and registered in the Iranian Registry of Clinical Trials (IRCT) center (IRCT20190711044176N1). The current research report has been adjusted to the CONSORT 2010 checklist.

### 2.2. Participants

Inclusion criteria were as follows: married women, 18–49-year-old nonpregnant with signs and symptoms of VVC (including cheese-shaped and adhesive vaginal discharge, inflammation (redness) and itching, and fungal culture confirmed VVC infection), not period of bleeding menstrual cycle, no breastfeeding, no known medical conditions such as diabetes or immunodeficiency, no immunosuppressive drugs, no vaginal drugs or douches within 48 hours prior to the study, no history of recurrent VVC, no sexual intercourse in the past 24 hours, without related Trichomonas vaginitis, bacterial vaginitis or cervicitis and PID, without herbal or chemical antifungal medications related to the treatment of genital infections in the last 10 days, no previous allergic reaction to honey, *N. sativa* and clotrimazole, no history of curettage, hysterosalpingography or uterine surgery, blood transfusion, or chemotherapy in the past two weeks.

The exclusion criteria were as follows: pregnancy, the patient does not want to continue participating, mistakenly taking a medical prescription, and simultaneous use of antibiotics.

### 2.3. Sample Size

At a significance level of 0.05, test power of 80%, and a significance level of 20% reduction in redness, itching, and discharge the sample size was estimated to be 35 women per group after quantification according to the following formula. Assuming a 20% drop-out rate, a sample size of 42 patients in each group was calculated. The sample size was determined based on the previous research [23].

(1) $$\begin{array}{c} n=\frac{{\left[Z_{1}-\sqrt[{\alpha /2}]{2\bar{p}\bar{q}}+Z_{1}-\sqrt[{\beta }]{\left(p_{1}q_{1}+p_{2}q_{2}\right)}\right]}^{2}}{\left(p_{1}-p_{2}\right)},p_{1}=1,q_{1}=0,p_{2}=0.8,q_{2}=0.2\text{,}\\ \bar{p}=\frac{p_{1}+p_{2}}{2}=0.9\text{,}\\ n=\frac{{\left[1.96\sqrt{2\times 0.9\times 0.1}+0.84\sqrt{\left(0+0.16\right)}\right]}^{2}}{{\left(1-0.8\right)}^{2}}=35\text{.} \end{array}$$

### 2.4. Statistical Analysis

Data was analyzed times using SPSS software version 16.0 (SPSS Inc., Chicago, IL, USA). A *p* value less than 0.05 was considered statistically significant. The mean and standard deviation were used for quantitative variables, and frequency table was applied for qualitative variables. Assuming the normality of the data, the independent statistics *t*-test, chi-square test, and Fisher's exact test were used for analysis.

### 2.5. Randomization

Initially, convenience sampling was used for this study, and then 84 women with VVC were assigned to the *N. sativa*-honey group or the 1% clotrimazole group through the random block allocation method based on the individual code at a 1 : 1 ratio. This method was used with 4 and 6 blocks. This process continues until the sample size was completed.

The participants, researcher, and analyst responsible for the randomization were not aware of the group assignment for participants.

### 2.6. Intervention

Participants with positive yeast cultures were randomly divided into two main groups: (1) 39 women received 1% clotrimazole vaginal cream and (2) 40 women received *N. sativa*-honey vaginal cream. Clotrimazole vaginal cream (1%) was purchased from ParsDaru Co., Tehran, Iran (batch number: 890037) and *N. sativa*-Honey vaginal cream was prepared in the Pharmacy Laboratory of the School of Persian Medicine, Tehran University of Medical Sciences, Tehran, Iran. 30% honey and 3% *N. sativa* extract were used to prepare the vaginal cream. Both the groups of cream tubes were similar in shape, size, and weight (50 grams). All tubes which were coded A or B and randomly given to the patients. The participants must apply an applicator (5 grams) of cream before going to the bed for seven nights.

### 2.7. Outcome Measures

A questionnaire survey was used to collect demographic and economic data from the participants. A checklist was provided to study participants to record the daily use of vaginal cream. The symptoms and signs of VVC (redness, discharge, itching, and fungal culture) were investigated in the questionnaire with short questions (yes/no). The treatment effects, including clinical signs and symptoms and yeast culture, were surveyed by short questions (yes/no) 6–10 days after the end of the treatment course.

The drug complaint questionnaire was used to assess possible side effects 6–10 days after the end of the treatment course, and short questions (yes/no) were attached. Satisfaction with the drug and treatment method was evaluated using the drug satisfaction questionnaire 6–10 days after the end of the treatment course, and short questions were asked ((satisfied/unsatisfied)/(very/somewhat)).

### 2.8. Laboratory Procedures

Clinical and laboratory examinations were performed on eligible participants suspected of VVC who provided informed consent. At first, the subjects were placed in the dorsal lithotomy position with an empty bladder. Then, a disposable speculum was inserted into the vagina without impregnating lubricant materials. A clinical examination was performed to evaluate the cervix and vagina for redness and discharge (shape, color, consistency, and odor) and rule out other concurrent infections.

Vulvovaginal candidiasis was confirmed by laboratory examinations from the middle two-thirds of vaginal sidewall secretions, such as wet preparations (with physiological saline and 10% potassium hydroxide), PH assessment, Gram staining, and culture in Sabourad's Dextrose Agar with Chloramphenicol medium and clinical signs and symptoms (redness and discharge) by examination and expression of the itching by patients [10, 24]. The PH test paper was applied to measure vaginal acidity. *pH* of 4 to 4.5 was included in this study.

The first sterile swab was drawn on two glass slides and made into a wet preparation (physiological saline and 10% potassium hydroxide), which can not only identify yeast cells and mycelium, but also exclude other conditions, such as bacterial vaginosis (clue cells) and trichomoniasis (trichomonads). The second sterile swab on the third glass slide was spread to perform a Gram stain test to identify yeast cells or hyphae. Finally, a third sterile swab was used to collect a vaginal discharge sample and placed it on a Sabourad's Dextrose Agar plate containing chloramphenicol medium. The medium was tested daily for up to two weeks and, if a *Candida* colony formed, it was considered positive.

The mycologist, who did not know the previous results, examined the samples through a microscope. A positive culture result confirmed the diagnosis.

During the study, the subjects were followed up by phone to emphasize the timely use of medications and to check for any unexpected side effects and the date of the next visit. In addition, a follow-up visit was arranged 6–10 days after completion of the treatment. All women who reported positive laboratory results or signs and symptoms of VVC during follow-up received conventional medication (clotrimazole vaginal cream).

## 3. Results

**Figure 1:**
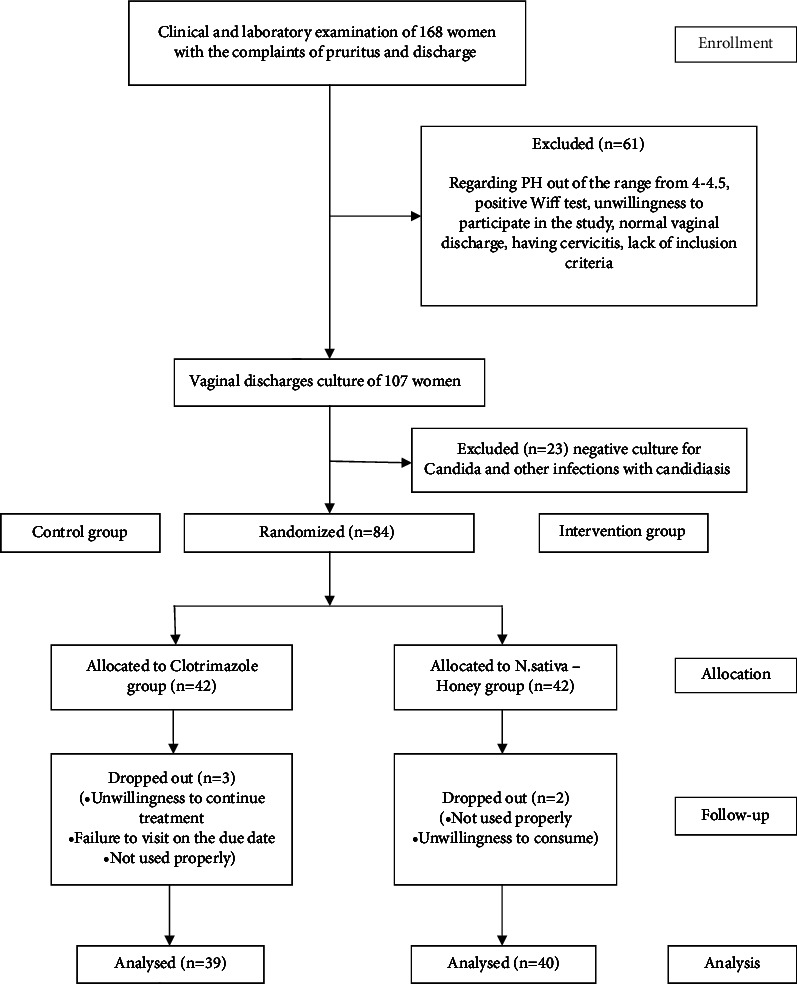
CONSORT flowchart of the study. Comparison between the efficacy of *Nigella sativa*-honey and clotrimazole on vulvovaginal candidiasis: a randomized clinical trial.

The current research was conducted from August 2019 to February 2020. Eighty-four eligible women were participated in the study. They were randomly divided into two groups, 42 women in the *N. sativa*-honey group and 42 women in the clotrimazole group were placed. Eighty-four eligible women had positive cultures. Until the end of the study, three women in the intervention group and 4 women in the control group withdrew from the study. At last, 79 participants completed the 7-day treatment period (Figure 1 is based on the CONSORT flow chart). Before treatment, the two groups had no significant differences in personal characteristics, obstetrics and medical history, health information, and body mass index (*p* > 0.05) (Table 1).

At the start of the study, 100% of the participants in both the treatment groups had discharge, redness, itching, and positive vaginal culture (Table 2). Compared with before treatment, the secretions, redness, itching, and fungal culture of the two groups improved in 6–10 days after treatment (*P* < 0.001) (Table 2). In the *N. sativa*-honey vaginal cream group, itching (*p* = 0.013) and discharge (*p* = 0.025) 6 to 10 days after treatment were statistically better than in the clotrimazole vaginal cream group (Table 2). Vaginal cultures of *Candida* in 29 (74.4%) subjects in the control group and 27 (67.5%) subjects in the intervention group became negative (Table 2).

There was no statistically significant difference between the two groups in terms of fungal culture results (*p* = 0.622), redness (*p* = 0.201), satisfaction with the method of drug used (*p* = 0.474), and satisfaction with the drug itself (*p* = 0.241) (Table 3). None of the groups showed any specific side effects.

## 4. Discussion

The current study aimed to evaluate and compare the efficacy of *N. sativa*-honey vaginal cream (traditional treatment) and clotrimazole vaginal cream (standard treatment) on VVC. This research is based on the assumption that *N. sativa* and honey can be an effective remedy for VVC [17, 22]. The current study was conducted in married, nonpregnant women of childbearing age (18–49) at the Baharloo Hospital of Tehran University of Medical Sciences, Tehran, Iran. The results of this research showed that all symptoms and signs of VVC in the two groups have been reduced, but in the intervention group, itching and secretions were significantly improved 6 to 10 days after treatment(Table 2).

The positive impact of *N. sativa* and honey products has been shown in several clinical trials [17, 22, 24] but there was no report in the literature regarding the impact of *N. sativa*-honey combination vaginal cream on VVC. The study of Banaeian et al. demonstrated that 70% honey to recover VVC symptoms but smaller than that of clotrimazole [22], but in this research, the combination of honey and *N. sativa* improved itching and secretion more than clotrimazole. In this study, a ratio of hydroalcoholic extract of 3% *N. sativa* and 30% honey were used in the preparation of vaginal cream. The explanation for this difference may be related to the additional influence of the combination of *N. sativa* and honey, which has also been seen in the study by Javadi et al. [21]. In fact, in our research, *N. sativa* seems to have a synergistic effect. Honey (30–70%) inhibits the growth of *Candida* [25]. The antifungal effect of honey may be due to the low *pH*, the osmotic pressure of the honey and the production of hydrogen peroxide and other factors [26]. Most of the biological and medicinal effects of *N. sativa* on the prevention of fungal growth and the effective anti-inflammatory and analgesic may be due to the presence of thymoquinone [18, 27]. The aim of this study was to evaluate the effectiveness of total extract not pure thymoquinone. However, in future studies, it is preferable to determine the amount of thymoquinone in the extract.

The results of this study are consistent with those of Saghafi et al. in the treatment of itching and secretion. In the study of Saghafi et al., each vaginal suppository used a 1% hydroalcoholic extract of *N. sativa*.

In this research, the combination of honey and *N. sativa* improved itching and secretion more than clotrimazole, but in Rasooli et al.'s study, this effect is similar to that of clotrimazole. In Rasooli et al.'s study, a ratio of hydroalcoholic extract of 2% *cinnamon* and 30% honey in the preparation of vaginal cream was used [28].

In our study, the results of fungal culture after treatment are not similar to those of Saghafi et al., this difference may be related to the *Candida* species. The rate of redness after treatment in this study was also not similar to that of Saghafi et al., this difference may be due to different measurement instruments.

The main limitation of this research was the lack of financial resources. We did not use chromium agar medium to determine the type of *Candida* species due to the high cost.

Further research is needed to evaluate impacts of *N .sativa*-honey and clotrimazole on the *Candida* species.

## 5. Conclusion

This study indicated the therapeutic effects of *N. sativa*-honey vaginal cream in improving some of the signs and symptoms of VVC. There are no side effects for women using *N. sativa*-honey vaginal cream. This complementary medicine appears to be safe, effective, and tolerable. However, more studies are needed to demonstrate safety and the mechanism responsible for *N. sativa*-honey vaginal cream.

## Figures and Tables

**Table 1:** The comparison of mean, standard deviation, and *p* value for study variables in two groups (*N. sativa*–honey and clotrimazole).

Variables	Clotrimazole (*n* = 39)	*N. sativa*-honey (*n* = 40)	*∗*_*p*_
Age (years); mean (SD)	33.10 (7.24)	32.65 (7.21)	0.782^†^

BMI; mean (SD)	26.34 (4.02)	26.66 (4.69)	0.750^†^

Education; *n* (%)			0.566^&^
High school	16 (41.0)	12 (30.0)	
Diploma	13 (33.3)	17 (42.5)	
Academic	10 (25.6)	11 (27.5)	

Job; *n* (%)			0.481^&^
Housewife	36 (92.3)	34 (85.0)	
Employee	3 (7.7)	6 (15.0)	

Husband education; *n* (%)			0.692^&^
High school	18 (46.1)	14 (35.0)	
Diploma	17 (43.6)	21 (52.5)	
Academic	4 (10.3)	5 (12.5)	

Economic status; *n* (%)			0.234^&^
Weak	11 (28.2)	6 (15.0)	
Moderate	26 (66.7)	29 (72.5)	
Good	2 (5.1)	5 (12.5)	

Menstrual cycle status; *n* (%)			0.808^††^
Normally	27 (69.2)	29 (72.5)	
Irregular	12 (30.8)	11 (27.5)	

Contraceptive method; *n* (%)			0.964^&^
OCP	4 (10.3)	4 (10.0)	
IUD	4 (10.3)	2 (5.0)	
Withdrawal	17 (43.6)	19 (47.5)	
Condom	5 (12.8)	6 (15.0)	
TL or vasectomy	3 (7.7)	4 (10.0)	
Nothing	6 (15.4)	5 (12.5)	

Anemia; *n* (%)			0.086^††^
No	35 (89.7)	28 (70.0)	
Yes	4 (10.3)	12 (30.0)	

Underwear material; *n* (%)			0.138^&^
Linen	34 (87.2)	29 (72.5)	
Plastic	0 (0.0)	3 (7.5)	
Both	5 (12.8)	8 (20.0)	

Use the pool; *n* (%)			0.316^††^
No	28 (71.8)	27 (67.5)	
Yes	11 (28.2)	13 (32.5)	

The number of times of intimacy with spouse per week; *n* (%)			0.185^&^
0	3 (7.7)	5 (12.5)	
1	12 (30.8)	8 (20)	
2	13 (33.3)	11 (27.5)	
3	7 (17.9)	4 (10.0)	
≥4	4 (10.3)	12 (30.0)	

Days of vaginal culture; *n* (%)			0.327^&^
6th	14 (35.9)	13 (32.5)	
7th	22 (56.4)	19 (47.5)	
8th	2 (5.1)	2 (5.0)	
9th	0 (0.0)	1 (2.5)	
10th	1 (2.6)	5 (12.5)	

^*∗*^Significance level: *P* < 0.05; ^†^Independent sample *t*-test;^††^Pearson's chi-square test; ^&^Fisher exact test; SD: Standard deviatio; BMI: body mass index.

**Table 2:** The comparison of the effects of *N. sativa*–honey and clotrimazol on VVC.

Variables	Clotrimazole (*n* = 39)		*N. sativa*- honey (*n* = 40)		*∗*_*p*_	Odds ratio (95% CI)
	No or negative	Yes or positive	No or negative	Yes or positive		
Abnormal discharge; *n* (%)					0.025^††^	5.7 (1.14, 28.38)
Before	0 (0.0)	39 (100)	0 (0.0)	40 (100)		
After	30 (76.9)	9 (23.1)	38 (95.0)	2 (5.0)		

Redness; *n* (%)					0.201^&^	4.45 (0.475, 41.797)
Before	0 (0.0)	39 (100)	0 (0.0)	40 (100)		
After	35 (89.7)	4 (10.3)	39 (97.5)	1 (2.5)		

Itching; *n* (%)					0.013^††^	3.942 (1.342, 11.575)
Before	0 (0.0)	39 (100)	0 (0.0)	40 (100)		
After	23 (59.0)	16 (41.0)	34 (85.0)	6 (15.0)		

Vaginal culture for candidiasis; *n* (%)					0.622^††^	0.716 (0.27, 1.902)
Before	0 (0.0)	39 (100)	0 (0.0)	40 (100)		
After	29 (74.4)	10 (25.6)	27 (67.5)	13 (32.5)		

^*∗*^Significance level: *P* < 0.05; ^&^Fisher Exact Test; ^††^Pearson's chi-square test; CI = confidence interval.

**Table 3:** The comparison of the satisfaction of *N. sativa*-honey and clotrimazole on VVC.

Satisfaction	Clotrimazole (*n* = 39)	N. sativa- honey (*n* = 40)	*∗*_*p*_
Satisfaction with the method of treatment; *n* (%)			0.474^††^
Very satisfied	25 (64.1)	29 (72.5)	
Somewhat satisfied	14 (35.9)	11 (27.5)	

Satisfaction with treatment; *n* (%)			0.241^††^
Very satisfied	23 (59.0)	29 (72.5)	
Somewhat satisfied	16 (41.0)	11 (27.5)	

^*∗*^Significance level: *P* < 0.05; ^††^Pearson's chi-square test.

## Data Availability

The datasets used during the current study are available from the corresponding author on reasonable request.

## Abbreviations

VVC: Vulvovaginal candidiasis
RVVC: Recurrent vulvovaginal candidiasis.

## Supplementary Materials

Supplementary Materials CONSORT 2010 checklist is included when reporting a randomized clinical trial.
